# Detailed Analysis of the Influencing Parameters on the Self-Healing Behavior of Dynamic Urea-Crosslinked Poly(methacrylate)s

**DOI:** 10.3390/molecules24193597

**Published:** 2019-10-06

**Authors:** Marcus Abend, Stefan Zechel, Ulrich S. Schubert, Martin D. Hager

**Affiliations:** 1 Laboratory of Organic and Macromolecular Chemistry (IOMC), Friedrich Schiller University Jena, Humboldtstr. 10, 07743 Jena, Germany; marcus.abend@uni-jena.de (M.A.); stefan.zechel@uni-jena.de (S.Z.); ulrich.schubert@uni-jena.de (U.S.S.); 2 Jena Center of Soft Matter (JCSM), Friedrich Schiller University Jena, Philosophenweg 7, 07743 Jena, Germany

**Keywords:** self-healing polymers, dynamic covalent bonds, characterization of self-healing, poly(methacrylate)s

## Abstract

For this paper, the self-healing ability of poly(methacrylate)s crosslinked via reversible urea bonds was studied in detail. In this context, the effects of healing time and temperature on the healing process were investigated. Furthermore, the impact of the size of the damage (i.e., area of the scratch) was monitored. Aging processes, counteracting the self-healing process, result in a decrease in the mechanical performance. This effect diminishes the healing ability. Consequently, the current study is a first approach towards a detailed analysis of self-healing polymers regarding the influencing parameters of the healing process, considering also possible aging processes for thermo-reversible polymer networks.

## 1. Introduction

The consumption of non-renewable resources has been increasing significantly over the last few years. These raw materials are utilized in a non-efficient manner, primarily as energy sources. However, there is also a large demand in many other industrial fields. Consequently, humanity is withdrawing these raw materials from the environment faster than regeneration takes place. Last year, 2018, Earth Overshoot Day was on August 1. It indicates the day on which the world population has consumed the regenerative resources of the corresponding year [1]. It is noteworthy that, two decades ago, Earth Overshoot Day was later, on September 30 [1]. 

In order to guarantee an optimized ecological and economical handling, new materials are also required to be self-healing materials, for example. This special ability increases the longevity of the used materials [2,3,4]. 

Generally, self-healing materials are divided into extrinsic and intrinsic systems [3]. Extrinsic systems are characterized by an additionally added compound, which provides the ability for self-healing. If the material is damaged, this healing agent is released and can close and subsequently heal the defect [3,5,6]. Microcapsules as well as microvascular networks have been used for this purpose [5,7,8,9]. The disadvantage of this type of self-healing is that no healing can be observed when the healing agents are consumed. 

Intrinsic materials represent another way of designing self-healing materials [2]. They possess the ability of local multiple healing cycles due to the presence of reversible chemical bonds and physical interactions, respectively [2]. These reversible bonds can be either dynamic covalent bonds [10], such as the Diels–Alder reaction [11,12,13], or radical-based systems [14] as well as supramolecular interactions [15], such as ionic interactions [16], π–π interactions [17], metal ligand interactions [18,19,20,21,22], and hydrogen or halogen bonds [23,24,25,26,27]. Basically, polymers offer a variety of different possibilities to achieve the ability for healing. In the context of the current study, there are also studies regarding reversible urea and urethane moieties, which are implemented into polymeric structures [28,29]. In the case of ureas, sterically demanding groups were attached to the amine compound revealing reversibility of the formed urea product. Thus, a larger substituent results in a more instable urea moiety and in a more efficient opening of the bond [29]. This behavior could also be implemented into methacrylates resulting in polymers with high E-moduli, which are unusual for self-healing materials. Those materials featured a suitable self-healing behavior while featuring mechanical stiffness [30].

The current study examined the relationship between mechanical properties and healing parameters such as temperature, time, and damage area for a urea-crosslinked copolymer, evaluating the optimal healing conditions (highest efficiency under the most moderate conditions minimizing aging). For this purpose, temperature was used as a healing trigger, since the use of a sterically hindered amine can result in an opening of the urea motif under thermal treatment responsible for the mobility required for the healing [29,30]. Furthermore, the aging behavior of the polymers was analyzed in detail and correlated to the applied healing conditions in order to evaluate the optimal compromise between degradation and restoration of the material. 

## 2. Results and Discussion

**Scheme 1:**
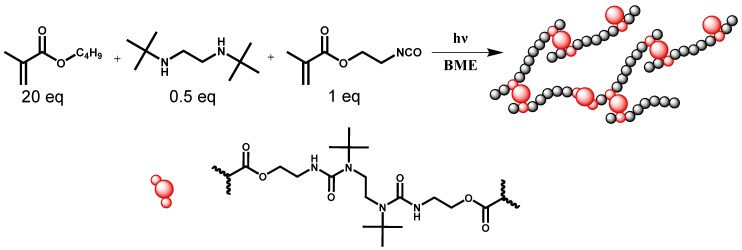
Schematic representation of the synthesis of the polymer network using a photo polymerization process with benzoin methyl ether (BME) as initiator and light between 320 and 520 nm.

The urea-crosslinked copolymers were synthesized according to Zechel et al. (Scheme 1) [30]. The procedure can be summarized as follows. The commercially available monomers butyl methacrylate and 2-isocyanate ethyl methacrylate as well as the diamine *N,N*’-di-*tert*-butylethylenediamine were mixed. The mixture was polymerized by adding the photoinitiator benzoin methyl ether (BME). Subsequent photopolymerization with the simultaneously occurring urea formation resulted in the formation of a dynamic covalently crosslinked copolymer network. The polymerization was performed in a special form to obtain directly a dog-bone tensile test specimen. After polymerization, a thermal annealing for 12 h was required in order to ensure a complete monomer conversion and polymerization.

**Figure 1:**
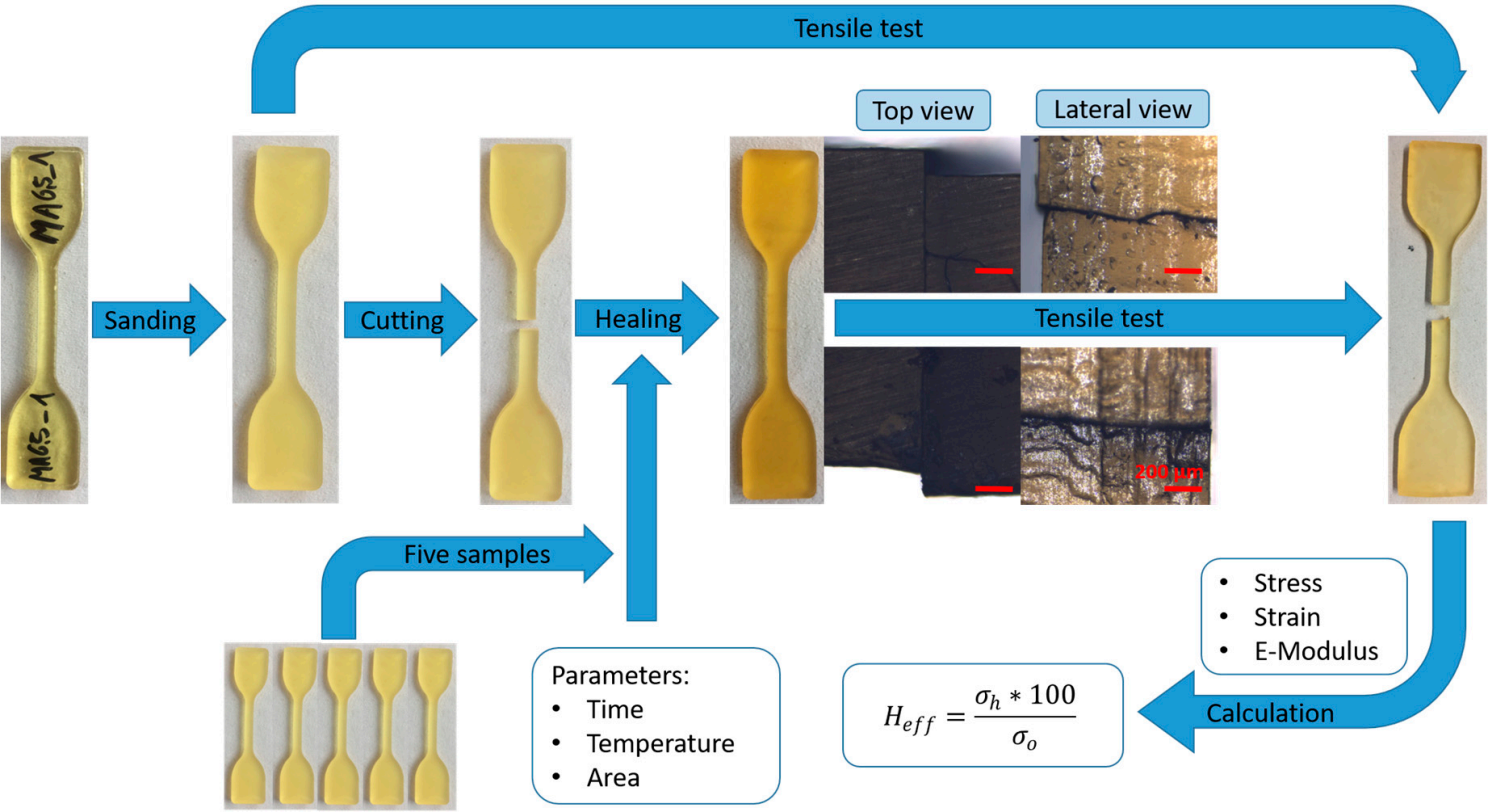
Schematic representations of the sample preparation and the measuring process. σ_o_ is determined from the original samples and σ_h_ from the healed sample. The healing efficiency is always considered regarding the different parameters.

**Figure A1:**
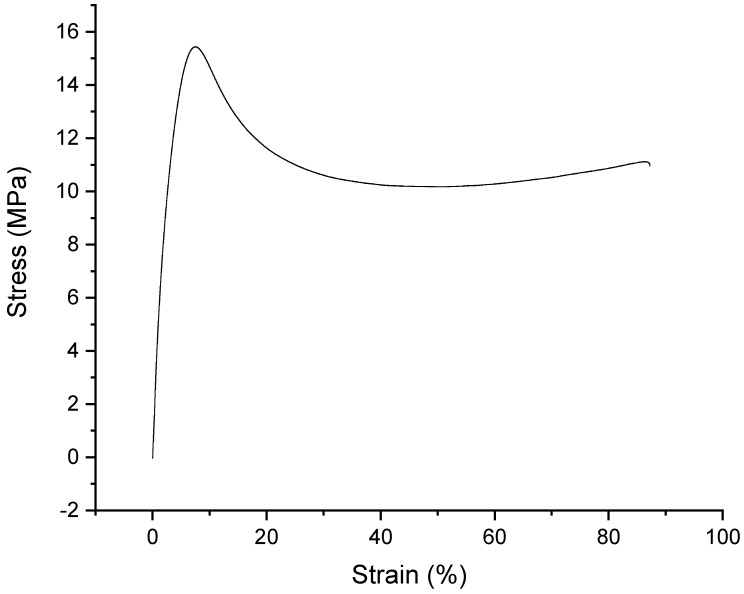
Stress–strain diagram of a tensile measurement of the urea-crosslinked copolymer network.

Figure 1 summarizes the workflow of the sample preparation and the subsequent measurements. All samples were sanded to level all inhomogeneous surfaces after the photopolymerization. The dimensions of the tensile specimens were then measured. All tensile tests were subsequently carried out for the original samples. An exemplary representation can be seen in Figure A1. The polymer shows a typical behavior of thermoplastics. Average yield strengths of 17 MPa and an E-modulus of 0.6 GPa were measured for the original samples. These values are in the range of the mechanical properties published by Zechel et al. [30]. In order to determine the self-healing, the samples were cut in the middle horizontally with a scalpel. The subsequent healing process was carried out according to the studied parameters. For each dependency, at least five samples were measured in the tensile test and the result represents the mean value of these measurements. After the measurement, the self-healing efficiency was determined according to the equation in the diagram (Figure 1). Additional microscopy images of the healed part of the polymer are also depicted in Figure 1. It becomes apparent that slight deviations from the original cross-sectional area may be present in bulk healing. The images shown are examples in order to better illustrate the cross-sectional area. For the tensile tests, only samples with a deviation of less than 5% were used. The deviations from the top view as well as from the side view were considered. A further analysis of this error cannot be carried out due to the large number of samples.

### 2.1. Time-Dependency of the Original Samples

**Figure 2:**
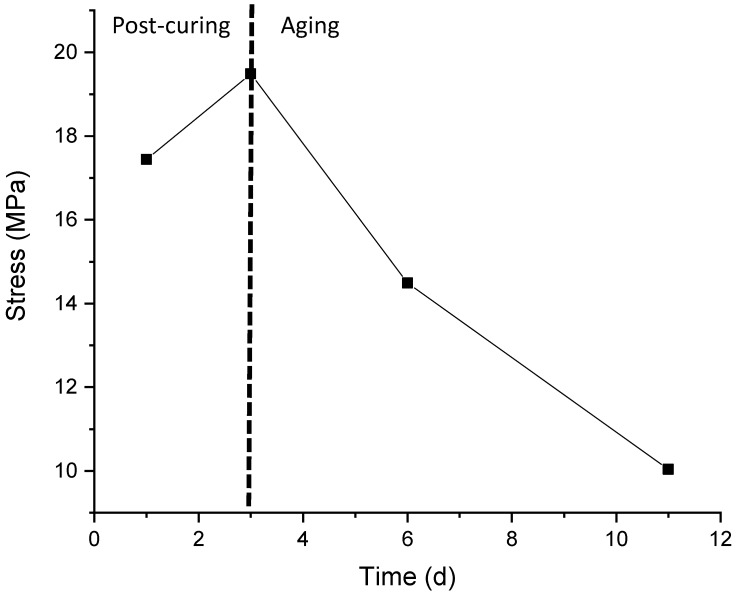
Dependency of stress value obtained by tensile test measurement on the annealing time (the average standard deviation is 1.69 MPa).

In order to observe the aging of this polymer, all samples were annealed at 100 °C for different times (Figure 2). It can be observed that the mechanical properties are still increasing after up to three days of annealing, which is presumably based on a post-curing effect resulting in more densely crosslinked networks. After three days, the stress values decrease significantly, which also has an influence on the healing efficiency later on. The decrease is caused by an aging process of the polymer network, since side reactions can occur in urea or urethane containing polymers in the case of long-term annealing [31,32]. This effect is presumably enhanced by the high reversibility of the sterically hindered urea bond.

**Figure 3:**
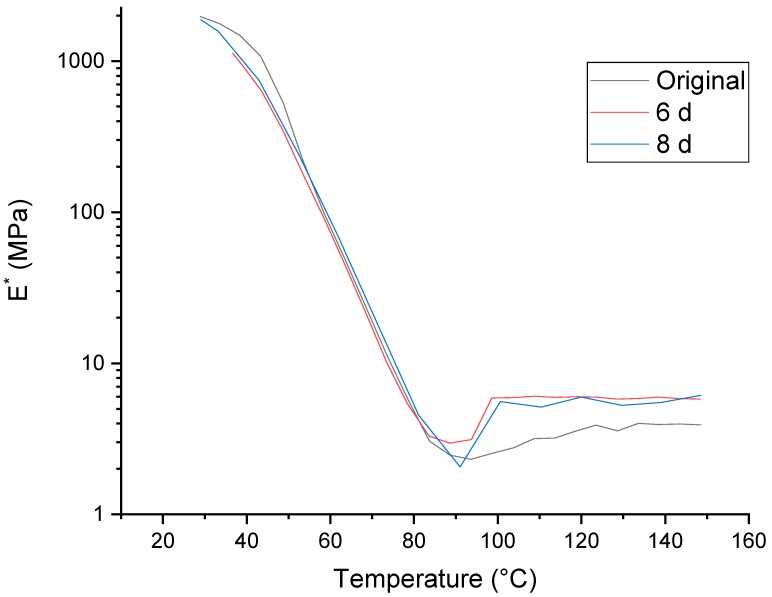
Dependency of the complex modulus of elasticity on temperature at different aging times.

**Figure A2:**
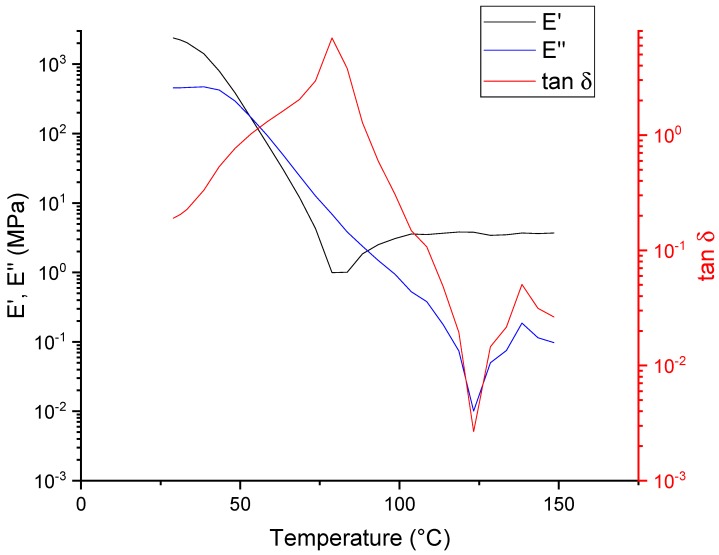
Results of the rheology and DMTA measurement of the urea-crosslinked copolymer network.

In order to evaluate the temperature-dependency behavior in more detail, small tensile samples were fabricated and measured by dynamic mechanical thermal analysis (DMTA). The results of a measurement are depicted in Figure A2. The complex elastic modulus depends on the thermal behavior of the polymer (see Figure 3). The glass transition temperature (*T*_g_ = 41 °C) determined by DMTA is in good agreement with the *T*_g_ = 30 °C obtained by differential scanning calorimetry (DSC) by Zechel et al. [30]. The glass transition temperature is slightly decreasing after 6 d (39 °C) and 8 d (37 °C) of annealing.

The main contribution of the *T_g_* value is based on the butyl methacrylate part, since 95% of the network are butyl methacrylate repeating units. The major degradation process is expected to originate from the reversible group (urea bond), which should have only a slight effect on the *T_g_*, because the opening temperature of the model systems (approximately 100 °C) is far above the *T_g_* [30].

### 2.2. Temperature-Dependency of the Healing Efficiency

**Figure 4:**
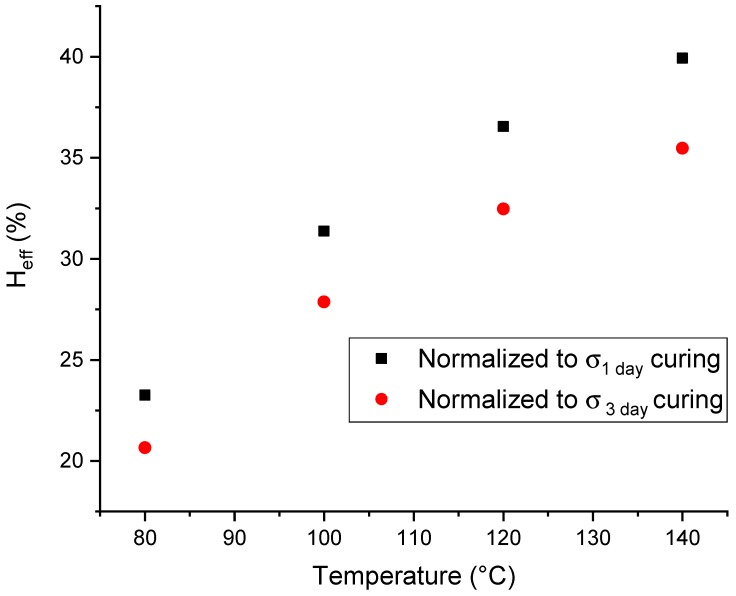
Temperature-dependency of healing efficiency. All measurements were performed after three days (the average standard deviation is SD_σ 1 day_ = 6.67% and SD_σ 3 day_ = 5.9%).

The investigation of the temperature dependency (all measurements were performed after three days) revealed a significant correlation between the applied temperature and the healing efficiency. An increasing temperature leads to a higher healing efficiency, that is, the yield strength is increased (see Figure 4 and Figure A4). This effect is based on the increased mobility of the polymer network due to the higher energy input. Furthermore, the reversibility of the urea bond is also enhanced with higher temperatures. However, the stability of the polymer must also be considered (see Figure 4). Zechel et al. have already measured TGA and DSC of the copolymer network [30]. These measurements revealed that the polymers are not stable above 140 °C. Therefore, a maximum healing temperature of 120 °C is recommended. The healing efficiencies are normalized to the yield strength of the corresponding annealing time and to the original yield strength. 

### 2.3. Time-Dependency of the Healing Efficiency

**Figure 5:**
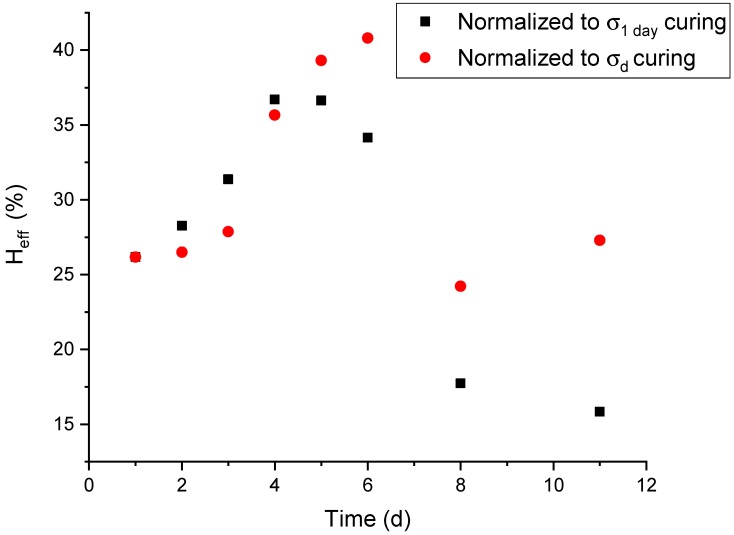
Time-dependency of the healing efficiency. All measurements were performed at 100 °C (the average standard deviation is SD_σ 1 day_ = 10.31% and SD_σd_ = 9.63%).

Besides the healing temperature, the healing time also has a significant impact on the achievable healing efficiency. Therefore, further testing to evaluate this parameter was performed. The samples were always healed at 100 °C for the corresponding periods of time. When considering the healing efficiency compared to the yield point of the untreated samples, it is clearly visible that the healing efficiency decreases significantly after six days (see Figure 5). Within the first four days, there is a continuous increase in the healing efficiency with a maximum reached after four days of healing. This behavior can be explained by the post-curing process described above. After a certain time, the aging is predominantly counteracting the restoration of the mechanical properties. For most self-healing polymers described in the literature higher, self-healing efficiencies are described for longer healing times. Additional effects have not been studied in detail.

If the healing efficiency is calculated using the yield stress of the original samples, which are also annealed for the corresponding time, the decrease in the healing efficiency starts after six days. Therefore, the aging process can also be differentiated. After six days, the aging seems to influence the general healing mechanism, resulting in a significant drop of the efficiency after 8 or 11 days of healing. The previous results were normalized to corresponding measured stresses. If no direct measurement of the stress was available, it was interpolated from Figure 2.

### 2.4. Area-Dependency of the Healing Efficiency

Self-healing is an interfacial process occurring at the polymer–polymer interface [33]. According to Kim and Wool, surface rearrangement, surface approach, and wetting are stages during the healing process [33]. Due to the importance of the interface, we wanted to study the influence of the size of the damaged area on the healing efficiency. To the best of our knowledge, a comparable study has as yet not been performed. 

Different factors may contribute to this correlation. An increased area contains a higher absolute amount of reversible bonds at the damage site. However, a uniform surface density of the reversible bonds can be expected. Small deviations in surface roughness, which lead to a surface contact that is not ideal, may have a larger impact for small damage areas. 

**Figure 6:**
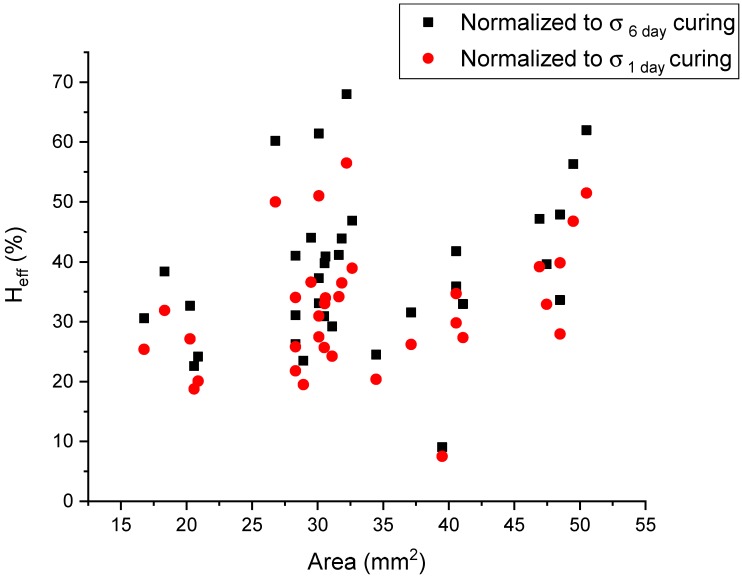
Area-dependency of the healing efficiency. All measurements were performed for six days at 100 °C (the average standard deviation is SD_σ 6 day_ = 17.14% and SD_σ 1 day_ = 13.76%).

For this purpose, samples with different cross-sectional areas were healed at 100 °C for six days. As can be seen in Figure 6, there is no clear dependency of damage area and the observed healing efficiency. The results vary significantly, particularly in the range of 27.5 to 40.0 mm^2^. There are various explanations for the large fluctuations within these results, for example, (A) inhomogeneities of the polymer composition due to the batch wise fabrication and (B) handling of the samples. The crack planes get a better contact for larger cross-sectional areas, which is the prerequisite for an efficient healing. The contact areas may not be ideal for the smaller areas, in particular due to the high E-modulus of the polymer (i.e., hard surfaces have to be pressed together).

## 3. Experimental Section

### 3.1. Materials and Methods 

**Figure A3:**
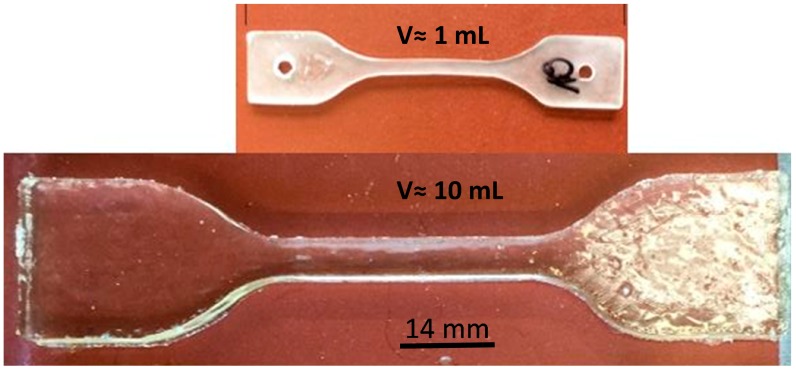
Picture of the dog-bone specimen of the urea-crosslinked copolymers. The upper small dog-bone represents a DMTA sample after the sanding process. The lower larger dog-bone represents a standard tensile sample before the sanding process.

**Figure A4:**
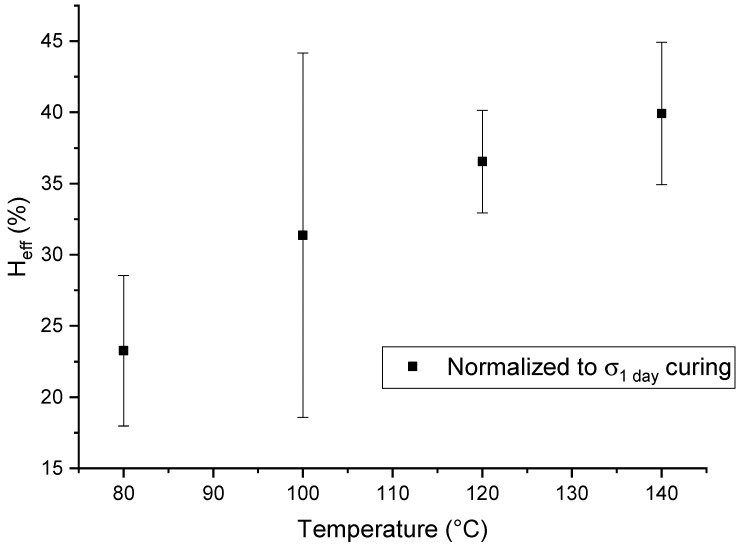
Temperature-dependency of healing efficiency with standard deviation. With increased temperature, a higher mobility is generated leading to higher healing efficiencies. All measurements were performed after three days.

The chemicals benzoin methyl ether (TCI, Eschborn, Germany; <98%), butyl methacrylate (Sigma-Aldrich, Darmstadt, Germany; 99%), *N,N*´-di-*tert*-butyl ethylene diamine (TCI; <98%) and 2-isocyanate ethyl methacrylate (abcr, Karlsruhe Germany; 98%) were used without further purification. All healing processes were performed in a drying oven (Heraeus Instruments, Hanau, Germany) at 100 °C. The mechanical scratches were inserted using a scalpel (BAYHA^®^ No. 23, Tüttlingen, Germany). The dynamic mechanical thermal analyses were carried out with the Gabo Qualimeter Eplexor^®^ 150 N (Selb, Germany). The samples were dynamically analyzed up to a maximum load of 10 N and an elongation of 0.25%. The heating rate was 2 K min^−1^. Tensile tests were performed on a standard tensile machine (Z020, Zwick/Roell, Ulm, Germany) at room temperature with a speed of two millimeters per minute for healed samples and with a speed of 5 mm per minute for standard samples. The two different sample forms were utilized and are illustrated in Figure A3. For each measurement condition, five different samples were produced and the mean value was determined.

### 3.2. Polymer Synthesis and Sample Preparation

The sample synthesis was performed according to Zechel et al. (see Scheme 1) [30]. Benzoin methyl ether was dissolved in BMA, *N,N*´-di-*tert*-butylethylenediamine, and 2-isocyanate ethyl methacrylate. The solution was thoroughly mixed and filled into the polymerization form. The solution was polymerized for 30 min using a UV chamber (Kulzer Dentacolor, Hanau, Germany). Afterwards, the molds with the formed polymer were stored overnight in the drying oven. This procedure ensured complete polymerization. This simple process made it easy to obtain the various sample shapes. The sample composition for the tensile samples can be found in Table 1. The tensile specimen form was produced in accordance with the specification for proportional tensile specimens (DIN EN 10002). The initial measurement length l_o_ corresponds to 5.65 times the square of the initial cross-section A_0_:

(1) $$l_{0}=k\sqrt{A_{0}}\;k\;=\;5.65.$$

In order to reduce the surface roughness, all samples were sanded. Therefore, commercially available abrasive paper from Siawat (Solingen, Germany) with different grain sizes (row: 40/80/120/200/1000/2000) was used. For all self-healing experiments, the samples were cut vertically with a scalpel on the constriction of the samples and brought into contact with a clamp aperture.

## 4. Conclusions

The influence of different parameters on the healing process of urea-crosslinked polymers was studied. Higher temperatures lead to higher healing efficiencies—healing coefficients of approximately 40% can be achieved at 140 °C. The healing time also has a strong influence on the obtainable healing efficiency. As is known from many other self-healing polymers, the healing efficiency increases with time for shorter healing times (<6 days) due to the reversible reaction, which is also accompanied by a post-curing process. However, after 6 days the effect is reversed due to an aging process occurring in the material. The mechanical properties decrease, leading also to lower healing coefficients. This effect is described here for the first time for self-healing polymers, which shows the importance of the applied conditions for the healing process. Lastly, it was revealed that the area of the cut has no significant influence on the healing coefficient.

## Figures and Tables

**Table 1:** The sample composition for the tensile samples.

Monomer	Tensile Test (mg)
Benzoin methyl ether	100
Butylmethacrylate	10,000
*N,N*´-di-*tert*-butylethylenediamine	302
2-isocyanate ethyl methacrylate	546
